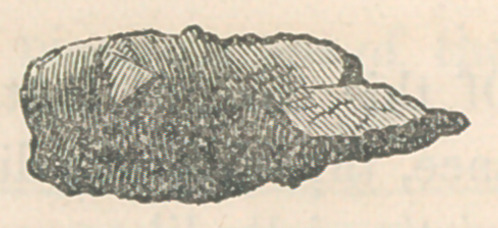# Removal of Salivary Calculus from Excretory Duct of Right Sub-Maxillary Gland

**Published:** 1870-10

**Authors:** Chas. M. Clark

**Affiliations:** Chicago


ARTICLE XXXIII.

### REMOVAL OF SALIVARY CALCULUS FROM EXCRETORY DUCT OF RIGHT SUB-MAXILLARY GLAND.

By CHAS. M. CLARK, M.D., Chicago.

History of Case.—*July 27th, 1870.*—Mr. S. came to my office, on the morning of July 22d, with, what he denominated, a *very sore throat*, accompanied by swelling underneath the tongue, which extended back on the right side, to the angle of the jaw, where quite a large-sized tumor could be defined. He had, occasionally, been subject to an enlargement under the angle of the right sub-maxillary, with considerable swelling under the tongue, for the past twenty years, but it had given him very little trouble or inconvenience.

It was most apt to occur at such times as he was conversing, or masticating his food, and then gave him some pain, until, by a peculiar manipulation with fingers and tongue, he reduced it.

The Symptoms—That were most prominent at the time he presented for treatment, were: excessive restlessness, some enlargement of right side of face; extreme sensitiveness when the parts were touched; increased flow of saliva and buccal mucus, of a ropy and adhesive nature; general febrile excitement; inability to swallow, but could manage to take a little water; mucous lining of floor of the mouth greatly enlarged and prominent, especially on the right side; temperature of the parts greatly increased, and a constant, dull, throbbing pain.

He stated that there was the sensation of *a* something drawing his tongue upwards and backwards, and completely filling the fauces; yet, there was no dyspnœa at any time, and I considered the matter to be an exaggeration, for I could depress the tongue, but not sufficiently to allow a free examination of the throat.

THE DIAGNOSIS—Of this case, at first, was "ranula;" but, on his second appearance, this idea was dismissed, for there was wanting the characteristic, jelly-like cyst. In its place was a fleshy tumor, highly inflamed, and giving to the touch a sense of fluctuation. I then pronounced it to be either an abscess or a calcareous deposit that was seeking an outlet.

THE TREATMENT—Given the case is as follows:

*July 22d*—Made application, to the parts, of sol. nit. arg. ℈ij to ℥j aqua, which gave relief. Later in the day, I freely incised the tumor and scarified the surrounding membrane. No pus appeared, although the surface was secreting a creamy exudation looking like pus. The hemorrhage from the part did good.

*July 23d*—Repeated the treatment at my office, and with the same results, he being able to swallow with more ease.

*July 24th*—Complained of feeling worse; more difficulty in deglutition, with increased febrile action. Desired that I should visit him at his home.

Ordered chlor. potass., sol. sulph. morph. and glycerine for gargle; full dose of soda et potass. tart.; also, toler nt doses of ant. et potass. tort., and, later in the day, gave full doses of "hydrate chloral" in aqua camphora, but it had no effect as a hypnotic, and I had recourse to morphia. During the afternoon, he suffered immensely; complained that he could not swallow, and that his tongue was being drawn up to the roof of his mouth. I gave him inhalations of chloroform, and applied it to the face externally, which gave prompt relief.

At 9 P.M., ordered a stimulating pediluvium and gr. xv. of Tulley's powder; also, put a bistoury into the tumor.

After this treatment, he was able to take a cup of gruel without much inconvenience.

*July 25th*—Discovered a hard point in the tumor beneath the tongue, and made a free incision, after which extracted a salivary calculus, weighing thirteen (13) grains, and composed, principally, of carbonate of lime. The following diagram will give an idea of the shape and size:

The extraction was followed by a small amount of pus.

The patient is an Englishman by birth, 36 years old, and an upholsterer by occupation, although for many years he pursued the avocation of a miner in Australia.

His voice, which has heretofore been harsh and coarse, is now as flexible and resonant as it was in his youth.

## Figures and Tables

**Figure f1:**